# 2-Dicyclo­hexyl­amino-3-phenyl-5,6-di­hydro-8*H*-thio­pyrano[4′,3′:4,5]thieno[2,3-*d*]pyrimidin-4(3*H*)-one

**DOI:** 10.1107/S1600536811009226

**Published:** 2011-03-15

**Authors:** Xizhen Liang, Yueming Zhou

**Affiliations:** a Faculty of Biology, Chemistry and Material Science, East China Institute of Technology, Nanchang 330013, Jiangxi, People’s Republic of China; b College of Chemistry and Chemical Engineering, Central South University, Changsha 410083, Hunan, People’s Republic of China

## Abstract

In the title compound, C_27_H_33_N_3_OS_2_, the dihedral angle between the two fused rings of the thieno[3,2-*d*]pyrimidine system is 3.73 (9)°. The phenyl ring is twisted with respect to the pyrimidine ring [dihedral angle = 71.60 (10)°] and the thio­pyran ring shows an envelope conformation with the S atom as the flap. An intra­molecular C—H⋯O inter­action occurs. In the crystal, inversion dimers linked by pairs of C—H⋯O hydrogen bonds occur.

## Related literature

For the properties of compounds containing the thieno­pyrimidine system, see: Santagati *et al.* (2002 ▶); Kikuchi *et al.* (2006 ▶). For related crystal structures, see: Hu *et al.* (2007 ▶); Xie *et al.* (2008 ▶).
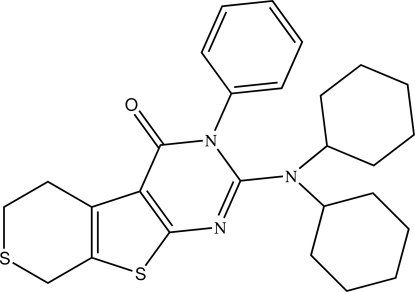


## Experimental

### 

#### Crystal data

C_27_H_33_N_3_OS_2_
*M* *_r_* = 479.70
Monoclinic, 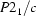

*a* = 10.7118 (10) Å
*b* = 22.601 (2) Å
*c* = 10.924 (1) Å
β = 109.939 (1)°
*V* = 2486.1 (4) Å^3^
*Z* = 4
Mo *K*α radiation
μ = 0.24 mm^−1^
*T* = 293 K
0.30 × 0.25 × 0.18 mm

#### Data collection

Bruker SMART CCD area-detector diffractometer
Absorption correction: multi-scan (*SADABS*; Bruker, 2007 ▶) *T*_min_ = 0.802, *T*_max_ = 0.874
12466 measured reflections
4365 independent reflections
3391 reflections with *I* > 2σ(*I*)
*R*_int_ = 0.027
Standard reflections: ?

#### Refinement

*R*[*F* ^2^ > 2σ(*F* ^2^)] = 0.039
*wR*(*F* ^2^) = 0.097
*S* = 1.00
4365 reflections
298 parameters
H-atom parameters constrained
Δρ_max_ = 0.25 e Å^−3^
Δρ_min_ = −0.26 e Å^−3^

### 

Data collection: *SMART* (Bruker, 2007 ▶); cell refinement: *SAINT* (Bruker, 2007 ▶); data reduction: *SAINT*; program(s) used to solve structure: *SHELXS97* (Sheldrick, 2008 ▶); program(s) used to refine structure: *SHELXL97* (Sheldrick, 2008 ▶); molecular graphics: *SHELXTL* (Sheldrick, 2008 ▶); software used to prepare material for publication: *SHELXTL*.

## Supplementary Material

Crystal structure: contains datablocks I, global. DOI: 10.1107/S1600536811009226/hb5802sup1.cif

Structure factors: contains datablocks I. DOI: 10.1107/S1600536811009226/hb5802Isup2.hkl

Additional supplementary materials:  crystallographic information; 3D view; checkCIF report

## Figures and Tables

**Table 1:** Hydrogen-bond geometry (Å, °)

*D*—H⋯*A*	*D*—H	H⋯*A*	*D*⋯*A*	*D*—H⋯*A*
C2—H2*B*⋯O1^i^	0.97	2.54	3.359 (3)	142
C11—H11⋯O1^ii^	0.93	2.57	3.199 (3)	126

Symmetry codes: (i) 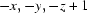; (ii) 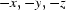.

**Fig. 1.**
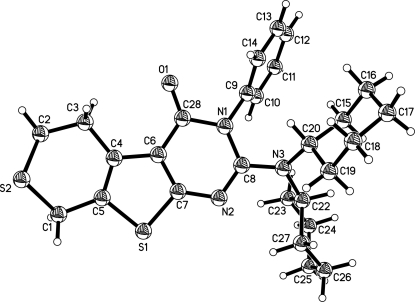
The molecular sturcture of (I) showing 50% displacement ellipsoids.

**Fig. 2.**
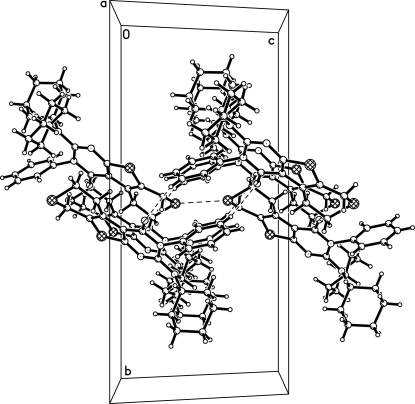
Packing diagram for (I) with C—H···O links shown as dashed lines.

## supplementary crystallographic information

### Comment

The derivatives of heterocycles containing thienopyrimidine system are of great importance because of their remarkable biological activity for use as potential drugs. They proved to show significant antifungal, antibacterial,antimicrobial, anticonvulsant and angiotensin antagonistic activities.Also some of these compounds show good antimalarial or potent multitargeted receptor tyrosine kinase inhibitive activities(Santagati *et al.*,2002; Kikuchi *et al.*,2006).In addition,the title compound have exhibited a high potential as efficient luminophores for liquid and fluorescent properties and markers in biological or supramolecular systems.some X-ray crystal structures of pyrimidinone derivatives have been reported(Hu *et al.*,2007; Xie *et al.*,2008). The bond lengths and angles are unexceptional. The thieno (A), the pyrimidinone (B) and the C9—C14 benzene(C), rings are, of course, planar and the dihedral angles between them are A/B = 3.73 (9) °, B/C = 71.60 (10) °. The thiopyrano ring in (I)(Fig1) shows a distored chairconformation [φ = 164.0 (3) ° and θ = 128.83 (18) °, Puckering Amplitude =0.6360 (18)%A].The weak intermolecular C—H···O hydrogen bonds (Table 1) link the molecules inti centrosymmetric dimmers (Fig 2).

### Experimental

To a solution of iminophosphorane (1.01 g, 2 mmol) in anhyd. CH_2_Cl_2_ (15 ml) aromatic isocyanate (2.2 mmol) was added under nitrogen atmosphere at room temperature. After the reaction mixture was left unstirred for 6–12 h at 0–5°C, the iminophosphorane had disappeared (TLC monitored). The solvent was removed off under reduced pressure and Et2O / petroleum ether (1:2, 20 ml)was added to precipitate triphenylphosphine oxide. Removal of the solvent gave carbodiimides, which were used directly without further purification. To the solution of in dichloromethane (15 ml) was added dialkylamine (2.2 mmol). After the reaction mixture was left unstirred for 4–6 h, the solvent was removed and anhyd. EtOH (15 mL) with several drops of EtONa in EtOH was added. The mixture was stirred for 6–12 h at room temperature. The solution was condensed and residue was recrystallized from EtOH to give yellow blocks of the title compound.

### Crystal data

**Table:**

C_27_H_33_N_3_OS_2_	*F*(000) = 1024
*M**_r_* = 479.70	*D*_x_ = 1.282 Mg m^−^^3^
Monoclinic, *P*2_1_/*c*	Mo *K*α radiation, λ = 0.71073 Å
Hall symbol: -P 2ybc	Cell parameters from 4365 reflections
*a* = 10.7118 (10) Å	θ = 2.0–25.0°
*b* = 22.601 (2) Å	µ = 0.24 mm^−^^1^
*c* = 10.924 (1) Å	*T* = 293 K
β = 109.939 (1)°	Block, yellow
*V* = 2486.1 (4) Å^3^	0.30 × 0.25 × 0.18 mm
*Z* = 4	

### Data collection

**Table:**

Bruker DIFFRACTOMETER TYPE????diffractometer	3391 reflections with *I* > 2σ(*I*)
Radiation source: fine-focus sealed tube	*R*_int_ = 0.027
graphite	θ_max_ = 25.0°, θ_min_ = 2.0°
Absorption correction: multi-scan (*SADABS*; Bruker, 2007)	*h* = −12→11
*T*_min_ = 0.802, *T*_max_ = 0.874	*k* = −17→26
12466 measured reflections	*l* = −12→12
4365 independent reflections	

### Refinement

**Table:**

Refinement on *F*^2^	Primary atom site location: structure-invariant direct methods
Least-squares matrix: full	Secondary atom site location: difference Fourier map
*R*[*F*^2^ > 2σ(*F*^2^)] = 0.039	Hydrogen site location: inferred from neighbouring sites
*wR*(*F*^2^) = 0.097	H-atom parameters constrained
*S* = 1.00	*w* = 1/[σ^2^(*F*_o_^2^) + (0.0373*P*)^2^ + 1.0362*P*] where *P* = (*F*_o_^2^ + 2*F*_c_^2^)/3
4365 reflections	(Δ/σ)_max_ < 0.001
298 parameters	Δρ_max_ = 0.25 e Å^−^^3^
0 restraints	Δρ_min_ = −0.26 e Å^−^^3^

### Special details

**Table:**

Geometry. All e.s.d.'s (except the e.s.d. in the dihedral angle between two l.s. planes) are estimated using the full covariance matrix. The cell e.s.d.'s are taken into account individually in the estimation of e.s.d.'s in distances, angles and torsion angles; correlations between e.s.d.'s in cell parameters are only used when they are defined by crystal symmetry. An approximate (isotropic) treatment of cell e.s.d.'s is used for estimating e.s.d.'s involving l.s. planes.
Refinement. Refinement of *F*^2^ against ALL reflections. The weighted *R*-factor *wR* and goodness of fit *S* are based on *F*^2^, conventional *R*-factors *R* are based on *F*, with *F* set to zero for negative *F*^2^. The threshold expression of *F*^2^ > σ(*F*^2^) is used only for calculating *R*-factors(gt) *etc*. and is not relevant to the choice of reflections for refinement. *R*-factors based on *F*^2^ are statistically about twice as large as those based on *F*, and *R*- factors based on ALL data will be even larger.

### Fractional atomic coordinates and isotropic or equivalent isotropic displacement parameters (Å^2^)

**Table:**

	*x*	*y*	*z*	*U*_iso_*/*U*_eq_	
S1	0.52363 (5)	0.09045 (3)	0.58192 (5)	0.05635 (17)	
S2	0.36688 (6)	−0.00416 (3)	0.84325 (5)	0.06459 (19)	
N2	0.39389 (15)	0.14368 (7)	0.35103 (15)	0.0446 (4)	
N1	0.17515 (14)	0.11220 (7)	0.23197 (14)	0.0381 (4)	
O1	0.06017 (13)	0.04696 (6)	0.31444 (12)	0.0520 (4)	
C6	0.28078 (18)	0.07129 (8)	0.44376 (17)	0.0392 (4)	
C7	0.38904 (18)	0.10337 (8)	0.44241 (18)	0.0414 (4)	
C4	0.30430 (18)	0.03617 (8)	0.55936 (17)	0.0408 (4)	
C8	0.28574 (17)	0.14842 (8)	0.24936 (17)	0.0382 (4)	
C5	0.43038 (19)	0.04268 (9)	0.64211 (18)	0.0456 (5)	
C3	0.2018 (2)	−0.00226 (10)	0.5853 (2)	0.0532 (5)	
H3A	0.1593	−0.0265	0.5091	0.064*	
H3B	0.1342	0.0228	0.5990	0.064*	
C1	0.4921 (2)	0.01507 (10)	0.7740 (2)	0.0568 (6)	
H1A	0.5547	0.0426	0.8312	0.068*	
H1B	0.5405	−0.0202	0.7664	0.068*	
C2	0.2587 (2)	−0.04225 (10)	0.7023 (2)	0.0587 (6)	
H2A	0.3078	−0.0740	0.6798	0.070*	
H2B	0.1862	−0.0599	0.7238	0.070*	
N3	0.27679 (15)	0.18922 (7)	0.15363 (15)	0.0417 (4)	
C9	0.08042 (18)	0.10101 (8)	0.10264 (17)	0.0392 (4)	
C22	0.39403 (19)	0.20341 (9)	0.11691 (19)	0.0455 (5)	
H22	0.3599	0.2280	0.0386	0.055*	
C20	0.16227 (19)	0.23014 (9)	0.11292 (18)	0.0455 (5)	
H20	0.0966	0.2139	0.1481	0.055*	
C10	0.12331 (19)	0.07079 (9)	0.01470 (18)	0.0447 (5)	
H10	0.2115	0.0589	0.0379	0.054*	
C14	−0.05072 (19)	0.11717 (9)	0.07028 (19)	0.0485 (5)	
H14	−0.0803	0.1362	0.1307	0.058*	
C27	0.5036 (2)	0.23963 (10)	0.2115 (2)	0.0563 (5)	
H27A	0.5426	0.2176	0.2918	0.068*	
H27B	0.4669	0.2760	0.2319	0.068*	
C23	0.4467 (2)	0.14866 (10)	0.0736 (2)	0.0576 (6)	
H23A	0.3750	0.1290	0.0065	0.069*	
H23B	0.4799	0.1217	0.1466	0.069*	
C13	−0.1378 (2)	0.10476 (11)	−0.0530 (2)	0.0595 (6)	
H13	−0.2263	0.1162	−0.0762	0.071*	
C12	−0.0948 (2)	0.07562 (11)	−0.1418 (2)	0.0597 (6)	
H12	−0.1538	0.0677	−0.2249	0.072*	
C15	0.0939 (2)	0.23351 (11)	−0.0339 (2)	0.0597 (6)	
H15A	0.0736	0.1939	−0.0692	0.072*	
H15B	0.1529	0.2519	−0.0731	0.072*	
C26	0.6108 (2)	0.25425 (11)	0.1533 (3)	0.0683 (7)	
H26A	0.5735	0.2798	0.0783	0.082*	
H26B	0.6822	0.2756	0.2172	0.082*	
C19	0.1943 (2)	0.29232 (9)	0.1691 (2)	0.0593 (6)	
H19A	0.2379	0.2901	0.2627	0.071*	
H19B	0.2542	0.3116	0.1322	0.071*	
C11	0.0352 (2)	0.05831 (10)	−0.1077 (2)	0.0533 (5)	
H11	0.0640	0.0381	−0.1673	0.064*	
C24	0.5580 (2)	0.16290 (11)	0.0206 (2)	0.0696 (7)	
H24A	0.5961	0.1262	0.0035	0.083*	
H24B	0.5211	0.1839	−0.0614	0.083*	
C25	0.6657 (2)	0.19973 (11)	0.1127 (3)	0.0735 (7)	
H25A	0.7126	0.1765	0.1892	0.088*	
H25B	0.7289	0.2110	0.0709	0.088*	
C16	−0.0336 (2)	0.26934 (13)	−0.0668 (2)	0.0789 (8)	
H16A	−0.0734	0.2729	−0.1606	0.095*	
H16B	−0.0959	0.2486	−0.0352	0.095*	
C17	−0.0084 (3)	0.33031 (13)	−0.0069 (3)	0.0926 (10)	
H17A	−0.0927	0.3501	−0.0222	0.111*	
H17B	0.0419	0.3532	−0.0491	0.111*	
C18	0.0670 (3)	0.32814 (12)	0.1378 (3)	0.0844 (8)	
H18A	0.0110	0.3106	0.1816	0.101*	
H18B	0.0886	0.3681	0.1704	0.101*	
C28	0.16231 (18)	0.07445 (8)	0.33148 (17)	0.0387 (4)	

### Atomic displacement parameters (Å^2^)

**Table:**

	*U*^11^	*U*^22^	*U*^33^	*U*^12^	*U*^13^	*U*^23^
S1	0.0443 (3)	0.0596 (4)	0.0528 (3)	−0.0111 (3)	0.0006 (2)	0.0144 (3)
S2	0.0742 (4)	0.0727 (4)	0.0432 (3)	−0.0018 (3)	0.0152 (3)	0.0092 (3)
N2	0.0419 (9)	0.0445 (9)	0.0433 (9)	−0.0061 (7)	0.0092 (7)	0.0063 (8)
N1	0.0373 (8)	0.0404 (9)	0.0349 (8)	−0.0025 (7)	0.0104 (6)	0.0018 (7)
O1	0.0453 (8)	0.0625 (9)	0.0443 (8)	−0.0166 (7)	0.0101 (6)	0.0039 (7)
C6	0.0411 (10)	0.0366 (10)	0.0386 (10)	−0.0021 (8)	0.0117 (8)	−0.0009 (8)
C7	0.0404 (10)	0.0397 (11)	0.0413 (10)	−0.0016 (9)	0.0103 (8)	0.0024 (9)
C4	0.0457 (11)	0.0351 (10)	0.0383 (10)	−0.0020 (8)	0.0100 (8)	0.0004 (8)
C8	0.0394 (10)	0.0370 (10)	0.0388 (10)	0.0004 (8)	0.0141 (8)	−0.0005 (8)
C5	0.0487 (11)	0.0398 (11)	0.0426 (10)	−0.0043 (9)	0.0079 (9)	0.0034 (9)
C3	0.0517 (12)	0.0558 (13)	0.0463 (12)	−0.0094 (10)	0.0093 (10)	0.0103 (10)
C1	0.0571 (13)	0.0532 (13)	0.0480 (12)	−0.0049 (11)	0.0022 (10)	0.0092 (10)
C2	0.0591 (13)	0.0567 (14)	0.0568 (13)	−0.0099 (11)	0.0152 (11)	0.0150 (11)
N3	0.0416 (9)	0.0401 (9)	0.0445 (9)	0.0000 (7)	0.0162 (7)	0.0072 (7)
C9	0.0384 (10)	0.0393 (10)	0.0369 (10)	−0.0018 (8)	0.0091 (8)	0.0029 (8)
C22	0.0475 (11)	0.0442 (11)	0.0483 (11)	−0.0031 (9)	0.0208 (9)	0.0034 (9)
C20	0.0482 (11)	0.0447 (11)	0.0460 (11)	0.0075 (9)	0.0193 (9)	0.0099 (9)
C10	0.0422 (10)	0.0426 (11)	0.0487 (11)	−0.0026 (9)	0.0147 (9)	−0.0026 (9)
C14	0.0404 (11)	0.0585 (13)	0.0469 (11)	0.0016 (10)	0.0151 (9)	0.0003 (10)
C27	0.0519 (12)	0.0535 (13)	0.0639 (13)	−0.0087 (10)	0.0202 (11)	−0.0068 (11)
C23	0.0582 (13)	0.0525 (13)	0.0680 (14)	−0.0024 (11)	0.0292 (11)	−0.0060 (11)
C13	0.0364 (11)	0.0755 (16)	0.0592 (13)	0.0009 (11)	0.0066 (10)	0.0043 (12)
C12	0.0542 (13)	0.0705 (16)	0.0438 (12)	−0.0097 (12)	0.0032 (10)	−0.0024 (11)
C15	0.0596 (13)	0.0700 (15)	0.0468 (12)	0.0126 (12)	0.0146 (10)	0.0122 (11)
C26	0.0562 (14)	0.0533 (14)	0.0955 (18)	−0.0121 (11)	0.0261 (13)	0.0016 (13)
C19	0.0732 (15)	0.0461 (12)	0.0663 (14)	0.0049 (11)	0.0339 (12)	0.0014 (11)
C11	0.0595 (13)	0.0543 (13)	0.0468 (12)	−0.0083 (11)	0.0190 (10)	−0.0084 (10)
C24	0.0741 (16)	0.0686 (16)	0.0815 (17)	0.0040 (13)	0.0466 (14)	−0.0009 (14)
C25	0.0535 (14)	0.0666 (16)	0.111 (2)	0.0017 (13)	0.0422 (14)	0.0132 (15)
C16	0.0654 (16)	0.100 (2)	0.0686 (16)	0.0253 (15)	0.0187 (13)	0.0350 (16)
C17	0.094 (2)	0.078 (2)	0.119 (3)	0.0398 (17)	0.0537 (19)	0.0499 (19)
C18	0.104 (2)	0.0562 (16)	0.109 (2)	0.0245 (15)	0.0563 (19)	0.0098 (16)
C28	0.0406 (10)	0.0377 (10)	0.0375 (10)	−0.0027 (9)	0.0130 (8)	−0.0020 (8)

### Geometric parameters (Å, °)

**Table:**

S1—C7	1.7286 (19)	C14—C13	1.381 (3)
S1—C5	1.745 (2)	C14—H14	0.9300
S2—C2	1.799 (2)	C27—C26	1.526 (3)
S2—C1	1.803 (2)	C27—H27A	0.9700
N2—C8	1.308 (2)	C27—H27B	0.9700
N2—C7	1.365 (2)	C23—C24	1.527 (3)
N1—C8	1.399 (2)	C23—H23A	0.9700
N1—C28	1.425 (2)	C23—H23B	0.9700
N1—C9	1.453 (2)	C13—C12	1.375 (3)
O1—C28	1.216 (2)	C13—H13	0.9300
C6—C7	1.372 (3)	C12—C11	1.370 (3)
C6—C28	1.435 (2)	C12—H12	0.9300
C6—C4	1.439 (2)	C15—C16	1.522 (3)
C4—C5	1.353 (3)	C15—H15A	0.9700
C4—C3	1.500 (3)	C15—H15B	0.9700
C8—N3	1.373 (2)	C26—C25	1.497 (3)
C5—C1	1.501 (3)	C26—H26A	0.9700
C3—C2	1.514 (3)	C26—H26B	0.9700
C3—H3A	0.9700	C19—C18	1.521 (3)
C3—H3B	0.9700	C19—H19A	0.9700
C1—H1A	0.9700	C19—H19B	0.9700
C1—H1B	0.9700	C11—H11	0.9300
C2—H2A	0.9700	C24—C25	1.498 (3)
C2—H2B	0.9700	C24—H24A	0.9700
N3—C22	1.478 (2)	C24—H24B	0.9700
N3—C20	1.478 (2)	C25—H25A	0.9700
C9—C14	1.376 (3)	C25—H25B	0.9700
C9—C10	1.379 (3)	C16—C17	1.509 (4)
C22—C23	1.502 (3)	C16—H16A	0.9700
C22—C27	1.513 (3)	C16—H16B	0.9700
C22—H22	0.9800	C17—C18	1.511 (4)
C20—C15	1.521 (3)	C17—H17A	0.9700
C20—C19	1.525 (3)	C17—H17B	0.9700
C20—H20	0.9800	C18—H18A	0.9700
C10—C11	1.377 (3)	C18—H18B	0.9700
C10—H10	0.9300		
			
C7—S1—C5	91.39 (9)	H27A—C27—H27B	108.1
C2—S2—C1	96.33 (10)	C22—C23—C24	111.83 (19)
C8—N2—C7	115.60 (16)	C22—C23—H23A	109.2
C8—N1—C28	122.99 (14)	C24—C23—H23A	109.2
C8—N1—C9	120.81 (14)	C22—C23—H23B	109.2
C28—N1—C9	114.86 (14)	C24—C23—H23B	109.2
C7—C6—C28	117.97 (17)	H23A—C23—H23B	107.9
C7—C6—C4	113.91 (16)	C12—C13—C14	120.5 (2)
C28—C6—C4	128.07 (17)	C12—C13—H13	119.8
N2—C7—C6	127.12 (17)	C14—C13—H13	119.8
N2—C7—S1	121.94 (14)	C11—C12—C13	119.9 (2)
C6—C7—S1	110.80 (14)	C11—C12—H12	120.0
C5—C4—C6	111.25 (17)	C13—C12—H12	120.0
C5—C4—C3	124.39 (17)	C20—C15—C16	110.26 (18)
C6—C4—C3	124.36 (16)	C20—C15—H15A	109.6
N2—C8—N3	120.91 (16)	C16—C15—H15A	109.6
N2—C8—N1	122.10 (16)	C20—C15—H15B	109.6
N3—C8—N1	116.99 (15)	C16—C15—H15B	109.6
C4—C5—C1	127.50 (18)	H15A—C15—H15B	108.1
C4—C5—S1	112.64 (14)	C25—C26—C27	111.91 (19)
C1—C5—S1	119.85 (14)	C25—C26—H26A	109.2
C4—C3—C2	113.29 (17)	C27—C26—H26A	109.2
C4—C3—H3A	108.9	C25—C26—H26B	109.2
C2—C3—H3A	108.9	C27—C26—H26B	109.2
C4—C3—H3B	108.9	H26A—C26—H26B	107.9
C2—C3—H3B	108.9	C18—C19—C20	109.8 (2)
H3A—C3—H3B	107.7	C18—C19—H19A	109.7
C5—C1—S2	110.84 (15)	C20—C19—H19A	109.7
C5—C1—H1A	109.5	C18—C19—H19B	109.7
S2—C1—H1A	109.5	C20—C19—H19B	109.7
C5—C1—H1B	109.5	H19A—C19—H19B	108.2
S2—C1—H1B	109.5	C12—C11—C10	120.1 (2)
H1A—C1—H1B	108.1	C12—C11—H11	119.9
C3—C2—S2	113.16 (15)	C10—C11—H11	119.9
C3—C2—H2A	108.9	C25—C24—C23	112.6 (2)
S2—C2—H2A	108.9	C25—C24—H24A	109.1
C3—C2—H2B	108.9	C23—C24—H24A	109.1
S2—C2—H2B	108.9	C25—C24—H24B	109.1
H2A—C2—H2B	107.8	C23—C24—H24B	109.1
C8—N3—C22	120.49 (15)	H24A—C24—H24B	107.8
C8—N3—C20	118.98 (14)	C26—C25—C24	111.5 (2)
C22—N3—C20	118.51 (15)	C26—C25—H25A	109.3
C14—C9—C10	120.40 (18)	C24—C25—H25A	109.3
C14—C9—N1	121.13 (17)	C26—C25—H25B	109.3
C10—C9—N1	118.38 (16)	C24—C25—H25B	109.3
N3—C22—C23	110.62 (16)	H25A—C25—H25B	108.0
N3—C22—C27	117.73 (16)	C17—C16—C15	111.7 (2)
C23—C22—C27	111.94 (17)	C17—C16—H16A	109.3
N3—C22—H22	105.1	C15—C16—H16A	109.3
C23—C22—H22	105.1	C17—C16—H16B	109.3
C27—C22—H22	105.1	C15—C16—H16B	109.3
N3—C20—C15	113.60 (16)	H16A—C16—H16B	107.9
N3—C20—C19	113.94 (17)	C16—C17—C18	112.1 (2)
C15—C20—C19	109.83 (18)	C16—C17—H17A	109.2
N3—C20—H20	106.3	C18—C17—H17A	109.2
C15—C20—H20	106.3	C16—C17—H17B	109.2
C19—C20—H20	106.3	C18—C17—H17B	109.2
C11—C10—C9	119.78 (19)	H17A—C17—H17B	107.9
C11—C10—H10	120.1	C17—C18—C19	111.8 (2)
C9—C10—H10	120.1	C17—C18—H18A	109.3
C9—C14—C13	119.21 (19)	C19—C18—H18A	109.3
C9—C14—H14	120.4	C17—C18—H18B	109.3
C13—C14—H14	120.4	C19—C18—H18B	109.3
C22—C27—C26	110.57 (18)	H18A—C18—H18B	107.9
C22—C27—H27A	109.5	O1—C28—N1	120.33 (16)
C26—C27—H27A	109.5	O1—C28—C6	126.36 (17)
C22—C27—H27B	109.5	N1—C28—C6	113.24 (16)
C26—C27—H27B	109.5		
			
C8—N2—C7—C6	−5.7 (3)	C20—N3—C22—C23	−138.37 (18)
C8—N2—C7—S1	179.04 (14)	C8—N3—C22—C27	−72.5 (2)
C28—C6—C7—N2	7.6 (3)	C20—N3—C22—C27	91.2 (2)
C4—C6—C7—N2	−174.80 (18)	C8—N3—C20—C15	−131.05 (19)
C28—C6—C7—S1	−176.68 (14)	C22—N3—C20—C15	65.0 (2)
C4—C6—C7—S1	0.9 (2)	C8—N3—C20—C19	102.2 (2)
C5—S1—C7—N2	174.90 (17)	C22—N3—C20—C19	−61.8 (2)
C5—S1—C7—C6	−1.09 (16)	C14—C9—C10—C11	2.0 (3)
C7—C6—C4—C5	−0.2 (2)	N1—C9—C10—C11	178.53 (17)
C28—C6—C4—C5	177.14 (19)	C10—C9—C14—C13	−2.4 (3)
C7—C6—C4—C3	179.31 (19)	N1—C9—C14—C13	−178.87 (18)
C28—C6—C4—C3	−3.4 (3)	N3—C22—C27—C26	−175.41 (18)
C7—N2—C8—N3	177.25 (16)	C23—C22—C27—C26	54.7 (2)
C7—N2—C8—N1	−3.5 (3)	N3—C22—C23—C24	173.60 (18)
C28—N1—C8—N2	10.6 (3)	C27—C22—C23—C24	−53.0 (2)
C9—N1—C8—N2	−155.50 (17)	C9—C14—C13—C12	1.2 (3)
C28—N1—C8—N3	−170.12 (16)	C14—C13—C12—C11	0.5 (4)
C9—N1—C8—N3	23.8 (2)	N3—C20—C15—C16	171.91 (19)
C6—C4—C5—C1	178.0 (2)	C19—C20—C15—C16	−59.2 (2)
C3—C4—C5—C1	−1.4 (3)	C22—C27—C26—C25	−55.9 (3)
C6—C4—C5—S1	−0.7 (2)	N3—C20—C19—C18	−171.88 (18)
C3—C4—C5—S1	179.85 (16)	C15—C20—C19—C18	59.4 (2)
C7—S1—C5—C4	1.02 (17)	C13—C12—C11—C10	−1.0 (3)
C7—S1—C5—C1	−177.80 (18)	C9—C10—C11—C12	−0.3 (3)
C5—C4—C3—C2	−10.5 (3)	C22—C23—C24—C25	52.1 (3)
C6—C4—C3—C2	170.07 (19)	C27—C26—C25—C24	55.1 (3)
C4—C5—C1—S2	−23.8 (3)	C23—C24—C25—C26	−53.0 (3)
S1—C5—C1—S2	154.80 (12)	C20—C15—C16—C17	55.8 (3)
C2—S2—C1—C5	49.27 (17)	C15—C16—C17—C18	−52.8 (3)
C4—C3—C2—S2	47.5 (2)	C16—C17—C18—C19	53.3 (3)
C1—S2—C2—C3	−64.11 (18)	C20—C19—C18—C17	−56.4 (3)
N2—C8—N3—C22	35.6 (3)	C8—N1—C28—O1	174.84 (17)
N1—C8—N3—C22	−143.67 (16)	C9—N1—C28—O1	−18.3 (2)
N2—C8—N3—C20	−127.97 (19)	C8—N1—C28—C6	−8.0 (2)
N1—C8—N3—C20	52.7 (2)	C9—N1—C28—C6	158.83 (16)
C8—N1—C9—C14	−119.1 (2)	C7—C6—C28—O1	176.52 (19)
C28—N1—C9—C14	73.7 (2)	C4—C6—C28—O1	−0.7 (3)
C8—N1—C9—C10	64.4 (2)	C7—C6—C28—N1	−0.4 (2)
C28—N1—C9—C10	−102.80 (19)	C4—C6—C28—N1	−177.64 (17)
C8—N3—C22—C23	57.9 (2)		

### Hydrogen-bond geometry (Å, °)

**Table:**

*D*—H···*A*	*D*—H	H···*A*	*D*···*A*	*D*—H···*A*
C2—H2B···O1^i^	0.97	2.54	3.359 (3)	142
C11—H11···O1^ii^	0.93	2.57	3.199 (3)	126

Symmetry codes: (i) −*x*, −*y*, −*z*+1; (ii) −*x*, −*y*, −*z*.
